# COVID-19 and Challenging Working Environments: Experiences of Black Sub-Saharan African (BSSA) Front-Line Health Care Professionals Amid of COVID-19 Pandemic in the English Midlands Region

**DOI:** 10.1007/s40615-023-01906-w

**Published:** 2024-01-17

**Authors:** Nyashanu Mathew, Pfende Farai, Mandu Stephen Ekpenyong

**Affiliations:** 1 Department of Nursing & Allied Professions, Nottingham Trent University, 50 Shakespeare Street, Nottingham, NG1 4FQ UK; 2 Department of Public Health and Nursing, Manchester Metropolitan University, All Saints Campus, M1 5GF, Manchester, UK

**Keywords:** COVID-19, Discrimination, Black Sub-Saharan Africans, Equality, Awareness

## Abstract

**Purpose:**

The impact of COVID-19 is challenging for many health and social care workers. The impact has been more felt by all ethnic groups, but during the course of its tenure, it has become more apparent that the black community has been affected more than others. They have been reported to suffer more fatalities from the pandemic compared to their white counterparts. Blacks are reported to make a significant percentage of health care workers. They are sometimes undervalued, lowly paid, with many on insecure contracts and experiencing professional inequality. This study sought to explore the challenges experienced by Black Sub-Saharan African (BSSA) front-line workers in health care during COVID-19 pandemic.

**Methodology:**

The study utilised an explorative qualitative approach (EQA). Forty research participants were recruited for the study. Semi-structured interviews were used to collect data through online platforms which included Zoom, WhatsApp and Teams. A thematic approach was used to analyse data.

**Results:**

Following data analysis, the research found that the research participants experienced undermining of expertise, lack of appreciation and unfair allocation of tasks and were overlooked for promotion and perceived as carriers of COVID-19.

**Conclusion:**

This group was over-represented in agency and self-employed roles. There is need for a strong government commitment to prevent discrimination through enacting a comprehensive legislation to support tackling the problem. Race equality training awareness needs to be rolled out into healthcare organisations and empower managers to deal with equality issues at work.

## Introduction

According to the latest statistics from the John Hopkins University, confirmed COVID-19 cases had exceeded 13.3 million worldwide with over 580,000 fatalities and more than 7.3 million recoveries by June 2020 [1]. In the UK, as of 26 June, a total of people tested positive to coronavirus stands at 311,965 and the number of people who died is 43,575 [2].

The effect of the pandemic on different ethnic groups is quite significant, and it is becoming more and more clear that ethnic minority groups, particularly blacks, are reported to suffer more fatalities from the coronavirus pandemic. This disproportionate burden of the disease among Black, Asian and Minority Ethnic (BAME) communities has been acknowledged by the World Health Organisation, as well as health authorities in the UK and the USA. The Office for National Statistics [3] reported that black males are 4.2 times more likely to die from COVID-19 than white males, while black females are 4.3 times more likely to die from COVID-19 than white females. There is also confirmed evidence that ethnic minority groups are more disadvantaged than their counterparts from the statistics. Blacks and migrant workers are reported to make up 60% of social care workers. They are undervalued and lowly paid, with many on insecure contracts and professional inequality [4].

The disproportionate impact of COVID-19 on the BAME workforce was not largely recognised due to the lack of recognition and acknowledgement of their work and their roles within the settings they are employed. When combined with the other inequalities that black people face, such as unfair allocation of tasks, being overlooked for promotion, racism and the undermining of expertise that they face, it is only then that it can be seen that blacks in the health and social care sector, and the wider workforce, have ultimately suffered the outcome of COVID-19. This is particularly important given the smaller numbers of BAME staff who make up the senior grades of non-medical staff and the ‘very senior manager’ grades. Among the National Health Service (NHS) medical staff, a higher percentage of junior doctors are from the BAME groups than senior doctors. BAME health workers are also more likely to work in social and care homes, with lower remuneration and potentially longer hours [5]. It is, therefore, plausible that, alongside the socio-economic consequence of their job positions, these social care workers may be more likely to have increased contact with COVID-19 cases.

The disproportionate high death rates reported among health care workers from the BAME communities appear to be only partially explained by age, gender, socio-demographic characteristics and underlying health conditions [6]. Another issue worth considering is whether health workers from BAME communities are more likely to face barriers in receiving timely COVID-19 tests which is critical to receiving timely treatment. This is in view of the existing evidence that BAME communities are less likely to report positive experience with respect to access to health care (including emergency health services) and timely attention [7, 8].

One of the things that COVID-19 had done is to expose the long-term inequalities in our society. COVID-19 has surfaced some of the unspoken pending issues within the health care sector over the years. Many people have been trying to get these issues recognised; however, there seems to have been no progress until the COVID-19 pandemic. It has become necessary to explore the potential contribution of other known inequalities, including racism experienced by health workers of BAME origin and reduced awareness of and access to protection during COVID-19, e.g. personal protective equipment (PPE), infection prevention training and remote working, as this might have contributed to higher mortality among BAME health workers. The high death rates amongst BAME groups were a shift from the previous trends in mortality rates in the UK pre-COVID-19 pandemic. The white community were more susceptible to high death rates from any other causes in comparison to the BAME groups.

## Methodology

### Settings and Participants

The study utilised an explorative qualitative approach (EQA) [9]. The idea was not to offer a final solution to the problem but to understand the extent of the problem. The study utilised narratives with BSSA front-line health and social care professionals to explore challenges during COVID-19 pandemic.

The study was designed and conducted in accordance with the Social Research Association’s ethical guidelines and research procedures. The study protocol was approved by the Nottingham Trent University Ethics Committee. Prior to commencing the study, the protocol was also reviewed by two external academics to determine its suitability for the intended study. The research participants were recruited from organisations involved in health and social care which included nursing homes, residential homes and domiciliary care.

Prior to recruiting the research participants, the researchers wrote letters to managers of nursing homes, residential homes and domiciliary care agencies inviting their BSSA front-line health and social care professionals to take part in the study. Only those professionals who expressed interest to take part in the research had their details passed on to the researchers to make further arrangements for the interviews.

Participation in studies should be voluntary and anonymous [10]. Participants in this research study retained anonymity and were voluntary. All the research participants were furnished with an information sheet about the research and were given the opportunity to ask questions prior to commencing the interview. A consent form was also given to the research participants to sign before taking part in the research. It gave them the right to withdraw from the study at any time without giving reasons. Individual interviews allowed for more in-depth exploration of sensitive issues. A schedule to guide the narratives of research participants was devised, and prompts were used to probe for more information where necessary. A total of forty research participants took part in the study. Interviews were stopped after the 40th interview when new data could not be generated anymore.

### Data Collection

Due to the restrictions on COVID-19 pandemic, interviews were conducted through online platforms which included Teams, Zoom and WhatsApp. The interviews lasted for 45 min. Interviews were semi-structured, allowing the front-line health professionals to lead the discussion and spontaneously raise topics of importance to them within their narratives. The interview schedule covered a broad range of topics relating to BSSA front-line health and social care professionals’ experiences of COVID-19 and working in challenging environments.

### Data Analysis

All interviews were recorded and transcribed verbatim, then analysed using NVivo software. This was meant to enable drawing out of key themes and discussions [11]. Transcripts were coded initially using an inductive approach to draw out key themes emerging from the primary data. Transcripts were then re-read in more detail by members of the research team to refine the initially developed themes. They were further coded to produce higher-level concepts emerging from the research. These codes were then checked by a second independent researcher who was not initially involved in the research. The interpretation of data was discussed during face-to-face meetings and agreed between members of the research team. Codes were cross-referenced to draw out common or contrasting examples and illustrative quotes to support the wider theories were used [12]. This type of analysis produces a rich qualitative description, detailing individual experiences and adding depth to inconclusive quantitative evidence on the COVID-19 pandemic and challenges experienced by BSSA front-line health and social care professionals.

### Ethical Consideration

All the research participants were given an information sheet detailing information about the research. Prior to taking part in the research, all the research participants signed consent forms which allowed them to withdraw from the research study at any time without giving any reason. All interview transcripts were anonymised, and the data was destroyed after the research was completed.

## Results

Following data analysis, the following themes were formed: undermining of expertise, being overlooked for promotion, lack of appreciation, unfair allocation of tasks, BAME being over-representation in agency and self-employed roles and being perceived as carriers of the corona virus.

1. Undermining of expertise

The research participants reported that they felt their expertise was not being given full respect as they felt that it was being undermined. They also reported that when anything good was being reported on news about nurses, they rarely featured in pictures compared to the mainstream society. This explains why when the pandemic started, there were fewer numbers of the BAME group being reported as at risk or having contracted COVID-19.

“*…Paramedics said they had no concerns, but I was really worried because I know the resident better than the paramedics. I don’t know if it was about my colour because the day nurse was white and when she rang them in the morning, they immediately advised self-isolation and then took him to hospital…*” A female general nurse.

“*you will never see us on the news, you will not see black nurses because we are in the pit we don’t have a voice and no one wants to know our expertise*” A male mental health nurse.

(2) Overlooked for promotion

The research participants felt that BSSA professionals lacked representation in leadership and upward promotion. They further reported that despite high qualifications, they were not being considered for promotion and had no alternative but to leave.

“*We have a lack of representation in leadership, that is why our needs are not prioritised*.” A female general nurse.

“*I have a so many qualifications up to Master’s level, but I could not get beyond a band 6 so I left to work as a self-employed nurse. Now the government has put policies in place to stop umbrella companies. It’s a way to force us back to into the NHS but there are no opportunities there.*” A male mental health nurse.

“*I have been a mentor to student nurses and then in a few years’ time they become my manager. It is heart breaking…*” A female occupational health nurse.

“*Sometimes it makes me feel ashamed that I am still a band 6. I studied 4 years of nursing diploma, BSc studies in counselling, health visiting and prescriber, MSc public health, Specialist course in Occupational Health to improve my chances but nobody cares…*” A female general nurse.

The general feeling was that the reason why they were side-lined for promotion was the same reason why they felt were not considered when it came to COVID-19.

(3) Lack of appreciation

The research participants reported that despite putting their best at work, the managers were not appreciative of their contribution. They also felt that when they took sick leave, they are seen as if they are running away from work, but this was not an issue with professionals from the mainstream community.

“*I nursed a resident at end of life. I looked after him until he died and gave my all to his care. His family were so appreciative and could see that I went above and beyond. When he died, they wrote a letter of thanks and to commend me to the manager. The manager didn’t even tell me. I heard from other staff.*” A female mental health nurse.

“*I went for a break in my car because we could go out during our break. I was reported for taking a nap in my own car. If I had driven away from the home and gone elsewhere no one would care. I was not given a policy or taken through any HR process. I waited to be contacted and never heard anything from the day the manager asked me to leave. I just moved on to agency…*” A male mental health nurse.

“*white people went off sick from the start of this thing [pandemic] they didn’t hesitate. We the black people carried on, but it’s not appreciated*” A female mental health nurse.

(4) Targeted for blame

The research participants felt that they were being targeted for blame compared to their counterparts from other ethnic groups.

“*Medication went missing and I was blamed for it no-one else. There was no investigation. My colleagues in the home were shocked and even vouched for my conduct. The NMC found no case to answer because I had done nothing wrong, but the manager did not care. I never heard anything, and I didn’t want to go back where I am not wanted.*” A male mental health nurse.

(5) Unfair allocation of tasks

The research participants reported unfair allocation of tasks resulting in black health care professionals being allocated difficult and sometimes potentially infectious cases. They were being sent to settings were the COVID-19 patients were admitted and due to fears of losing their jobs and knowing their views are not heard quietly succumbed.

“*They [black people] are being severely affected because they are stuck in frontline, ‘in the pit’. This is why they are vulnerable.*” A male support worker.

“*right now, where I’m working, black staff are given seats and desks together, separate to all the white staff. I don’t know why but we are in our own corner of the place just laugh… and do our work*” A female support worker.

“*prior to the report on vulnerability of black people to COVID-19 the Majority of black nurses… were working in COVID-19 wards and this is still going on in some places especially if a black nurse is coming through the agency*” A male mental health nurse.

(6) Agency work vulnerability

The research participants reported that most black health professionals are moving to work for agencies or self-employment due to the discrimination they receive in big care organisations where they may have wished to take a permanent position for future security. However, during the COVID-19 pandemic, they found their services in demand more as more and more of their white colleagues in permanent roles were taking time off or being placed in less risky settings.

“*Black Africans left NHS, care and permanent employment due to discrimination and non-progression*” A male general nurse.

“*It’s simple I don’t want to work with people who don’t appreciate me. If you are unappreciated as a team member you become jittery and prone to make mistakes. It’s not good for me and it’s not good for anyone else. I won’t do that to myself.*” A female general nurse.

“*I’m starting a new job…my manager accidentally left a voicemail on my phone because she didn’t hang up the phone…she was basically telling someone how she didn’t want me there and saying the names of people who she would have preferred to have… had white sounding names…and saying she was forced to take me…I don’t know if it is about my race but I will be the only black person in the department…I felt really hurt…*” A male support worker

(7) Perceived as carriers of COVID-19

The research participants reported that they were being perceived as carriers of COVID-19 and that it made them feel unwanted and discriminated against. They also reported feelings of distress because of how they were being perceived.

“*I took a call from a doctor who told me he was too anxious to go to work because there are too many black people there and he was frightened to be infected by them…*” A female general nurse.

“*I have heard people say that black people spread COVID-19, especially around the time of the Black Lives Matter protests. I thought to myself, I am black, and I am here on the frontline how do you think it makes me feel. White people were also protesting with us…*” A female mental health nurse.

## Discussions

Confidence of individual professionals in an organisation is strongly linked to the support they get from management and the wider general professional environment which includes the external multi-disciplinary team [13]. The research participants reported feelings of being undermined by fellow professionals in the field. They also felt that they were rarely featured as the face of the organisation when there is good news compared to their white counterparts. Institutional racism and discrimination take place in many organisations in the world and has become part of some organisational climate making it very difficult to challenge or address [14]. Undermining of fellow professionals exposed them to COVID-19, for example, where their clinical diagnosis of a possible COVID-19 case is ignored and attended to later following infection of others. It is worth noting that these workers were also parents, spouses, partners and caregivers to other family members who became vulnerable and contracted COVID-19. Despite the issue being a health scare, it became a battle of races. There is need to have clear strategies of dismantling institutional racism, discrimination and bias in organisations. This may help to protect vulnerable black health care professionals from perpetuated institutional discrimination. Such strategies start with defining and understanding the impact of racism, discrimination and bias from the perspectives of the affected individuals as opposed to the perspectives of the perpetrators [15]. Organisations need to have open dialogue with all workers on the importance of equality and recognition of all workers regardless of their colour creed or religion. Ending racism, discrimination and bias can have positive impact on the outcomes and climate of organisations especially in health and social care.

Promotion can motivate health care professionals to work hard and enhance their professional growth including the ability to achieve organisational outcomes [16]. Consequently, non-recognition of workers through promotion can also have a dire impact on the affected workers including organisational outcomes. The research participants reported lack of representation in leadership and upward promotion. They also felt that their high qualifications were not being recognised for promotion. Because of this lack of upward movement, black health care professionals have remained at the bottom and are always on the front line in times of pandemics like COVID-19. There is need to address the bias in workers’ promotion through a clear strategy that addresses the present skewed representation of black professionals in leadership roles [17]. Representation of all races in the organisational leadership roles can unite workers from both sides of the racial divide on a common purpose and view the organisation as universal leading to motivation and co-operation. There is need for the government to reward organisations embracing multi-racial promotion of its workforce through funding to support the initiative. This will in turn encourage other organisation to join in the initiative.

The lack of recognition and appreciation of workers has negative impacts especially on private life of the affected individuals [18]. The vast majority of those who lack appreciation for their work indicate that their job has a negative impact on their private life. Similarly, in this study, the research participants reported that despite putting their best at work, the managers were not appreciative of their contributions. Furthermore, they also felt that when they go off sick, they were viewed as running away from work. This lack of appreciation from management predominantly from white backgrounds caused alienation and lack of faith in the whole organisation by black health care professionals [19]. This can also force black health care professions to report for work during pandemic periods like COVID-19 when not well, making them vulnerable. Such a feeling can also invoke mental health stress on black health care professionals and possibly result in loss of working hours through sickness [20]. There is need for organisations to constantly seek feedback from all workers on how they feel valued in their current position. In seeking such feedback, there is need to feel safe from victimisation on the part of the affected black professionals and trust the employers to deal with these issues fairly. This will create a conducive atmosphere for meaningful dialogue. More importantly organisations need to undergo cultural reincarnation to accommodate and appreciate a diverse culture embedded in equality and non-discriminatory practices [21]. Such a culture can prevent alienation, mistrust, and despondence on black health care professionals.

Fear of being targeted for blame can cause a feeling of uneasy on the affected individual [22]. Furthermore, it can also cause mental distress and loss of working hours in situations where no interventions are available for workers. The research participants reported being targeted for blame compared to their white counterparts from the mainstream society. Such a feeling can breed mistrust and despondence on the blamed individuals. This can also make them more vulnerable in times of pandemics like COVID-19. As alluded to earlier, organisations need to open up a dialogue and understand the lived experiences of those feeling targeted for blame. The gathered information can be used to form the bases of intervention in preventing targeted blame through adopting inclusive and non-discriminatory practices [23]. More importantly organisations need to recognise the existence of the problem in order to tackle it.

Allocation of tasks at workplaces needs to be fairly distributed across all the workers as this may affect morale of different workers [24]. The research participants reported unfair allocation of duties. They cited being allocated difficult cases and sometimes potentially COVID-19 cases compared to their white counterparts. Such a practice disproportionately exposed black health care professionals to COVID-19 in addition to their high chances of vulnerability. This can also affect the mental health and well-being of black health care professionals given the surge of COVID-19 in health and social care workplaces at the time in question [25]. The reported skewed allocation of difficult duties to black health care professionals is also exacerbated by poor representation of black professionals in the management and leadership of many health and social care organisations [26]. It is therefore important to note that it is not enough to only try and address the unfair allocation of duties without changing the organisational culture of promoting mainly white professionals into management and leadership positions. As discussed earlier, there is need for clear initiatives to change the unfair culture of promotion in organisations and adopt a more inclusive and racially unbiased promotion culture.

Agency workers move from one place to another in search of short- and long-term contracts [27]. The nature of their contracts makes it very difficult for their voices to be heard. Most of the research participants reported that they left permanent employment due to unfair treatment putting themselves in a more vulnerable situation. They reported being given highly challenging tasks and sometimes potential COVID-19 cases compared to their white counterparts who were permanent. There is evidence of dissatisfaction by agency workers because of poor treatment they receive from management in organisations they work for [28, 29]. The plight of agency workers in health and social care needs to be addressed to make sure that they are treated fairly. Managers in health and social care settings need more training around fair treatment of workers regardless of their contract and race. Such training can reduce bias, discrimination and prejudice resulting in positive outcomes for both the workforce and service users.

Negative perceived perceptions and vulnerability of a social group connected to an untreatable condition can impact heavily on the health and well-being of the affected social group [30]. Such an impact is not new as was seen with human immuno-deficiency virus (HIV) and its disproportionate impact on BSSA communities leading to stereotyping from other communities [31]. The research participants reported being perceived as carriers of COVID-19 and that it made them feel unwanted, discriminated and distressed in workplaces. They also reported feelings of distress because of how they were being perceived. There is need to raise awareness among professionals and communities on the impact of stigmatising other people about a disease that can affect anyone. Such an awareness can be incorporated in the training of staff and community health promotion programmes. There is also need for a proactive occupational health system in health and social care to support professionals affected by mental health.

## Implications for Future Practice

There is need for organisations to recognise the existence of race inequality and discrimination in workplaces. Such a recognition will pave way for a commitment to get rid of the practice. Furthermore, the government need to pass binding legislations to counter racial discrimination in organisations and workplaces. Issues of race and discrimination can mask major issues within workplaces if not addressed.

## Limitation of the Study

This research sought to understand the lived experiences of Black Sub-Sahara African front-line health and social care professionals and used a qualitative approach. However, future research needs to use both qualitative and quantitative approaches to address different contextual issues.

## Concluding Comments

There is need for a strong government commitment to prevent discrimination through enacting comprehensive legislation to support tackling the problem. Race equality training awareness needs to be rolled out into organisations and empower managers to deal with race and equality at work. Management and leadership in work settings need to challenge unconscious biases that lead them to overlook such important trends as they can have a spiralling effect to other important decisions that are taken.

## Data Availability

Data is available upon request through the corresponding author subject to the fulfilment of data protection regulations of Nottingham Trent University in terms of sharing research data.

## Code Availability

N/A.
